# The evaluation of the delayed swollen breast in patients with a history of breast implants

**DOI:** 10.3389/fonc.2023.1174173

**Published:** 2023-07-05

**Authors:** Grace C. Keane, Alexandra M. Keane, Ryan Diederich, Kaitlyn Kennard, Eric J. Duncavage, Terence M. Myckatyn

**Affiliations:** ^1^ Division of Plastic and Reconstructive Surgery, Washington University School of Medicine, Saint Louis, MO, United States; ^2^ MidAmerica Plastic Surgery, Glen Carbon, IL, United States; ^3^ Division of Surgical Oncology, Washington University School of Medicine, Saint Louis, MO, United States; ^4^ Department of Pathology and Immunology, Washington University School of Medicine, Saint Louis, MO, United States

**Keywords:** breast implant, BIA-ALCL, BIA-SCC, BIA-DLBCL, breast cancer

## Abstract

Breast implants, whether placed for reconstructive or cosmetic purposes, are rarely lifetime devices. Rupture, resulting from compromised implant shell integrity, and capsular contracture caused by constriction of the specialized scar tissue that normally forms around breast implants, have long been recognized, and remain the leading causes of implant failure. It is apparent, however, that women with breast implants may also experience delayed breast swelling due to a range of etiologic factors. While a majority of delayed seromas associated with breast implants have a benign etiology, this presentation cannot be ignored without an adequate workup as malignancies such as breast implant associated anaplastic large cell lymphoma (BIA-ALCL), breast implant associated diffuse large B-cell lymphoma (BIA-DLBCL), and breast implant associated squamous cell carcinoma (BIA-SCC) can have a similar clinical presentation. Since these malignancies occur with sufficient frequency, and with sometimes lethal consequences, their existence must be recognized, and an appropriate diagnostic approach implemented. A multidisciplinary team that involves a plastic surgeon, radiologist, pathologist, and, as required, surgical and medical oncologists can expedite judicious care. Herein we review and further characterize conditions that can lead to delayed swelling around breast implants.

## 1 Introduction

Breast implants remain the most common implanted medical devices in plastic surgery operating rooms. Over 350,000 women underwent cosmetic breast augmentation in the Unites States in 2021, making it the second most popular aesthetic procedure next to liposuction (1). Breast implants also represent the most common form of post-mastectomy reconstruction for the 1 in 8 women in the United States who will be diagnosed with breast cancer during their lifetimes. Though breast implants are approved by the Federal Drug Administration (FDA) for the purposes of breast augmentation or reconstruction, they are not without risk. This has come to light more recently with the discovery of breast implant associated anaplastic large cell lymphoma (BIA-ALCL) and the considerable attention it has garnered over the last decade.

BIA-ALCL is a rare T-cell lymphoma that most often presents as a delayed seroma surrounding a textured breast implant. A mass originating in the implant capsule may develop concurrently, or as the sole finding, with most cases presenting 8-10 years post-implantation, with earlier and later cases also reported. The first case of anaplastic T-cell lymphoma in proximity to a saline-filled breast implant was described as early as 1997, although recent literature identifies a possible earlier case description in 1996 (2–4). The first FDA safety communication regarding breast implants in 2011 and the recognition of BIA-ALCL as a separate category of malignancy by the World Health Organization (WHO) in 2016 sparked heightened awareness (5). Shortly thereafter, the National Comprehensive Cancer Network (NCCN) published guidelines for the diagnosis and treatment of BIA-ALCL, emphasizing early intervention and surgical treatment (6). As of April 1, 2022, the FDA has received a total of 1,130 United States and global medical device reports (MDRs) of BIA-ALCL (7).

Despite its recent notoriety, BIA-ALCL represents only a small fraction of delayed complications associated with breast implants. Specifically, making an accurate diagnosis when a patient presents with delayed breast swelling can be challenging. Development of a swollen breast one or more years after implantation carries with it a lengthy differential diagnosis representing a wide range of potential morbidity and mortality. In addition to BIA-ALCL, other malignancies associated with breast implants have been recognized, including breast implant-associated squamous cell carcinoma (BIA-SCC) and breast implant associated diffuse large B-cell lymphoma (BIA-DLBCL). In this review, we focus on the etiology of a swollen breast that develops in a delayed manner following placement of breast implants. We expand upon each of the breast implant-associated malignancies, including a discussion on the varied presentation, etiology, diagnostic algorithm, findings, and treatment modalities for each disease. We also review common findings and treatment modalities for benign causes of delayed breast swelling, including infection, benign seroma, trauma, hematoma, double capsule, and capsular contracture.

## 2 Diagnostic evaluation

**Figure 1:**
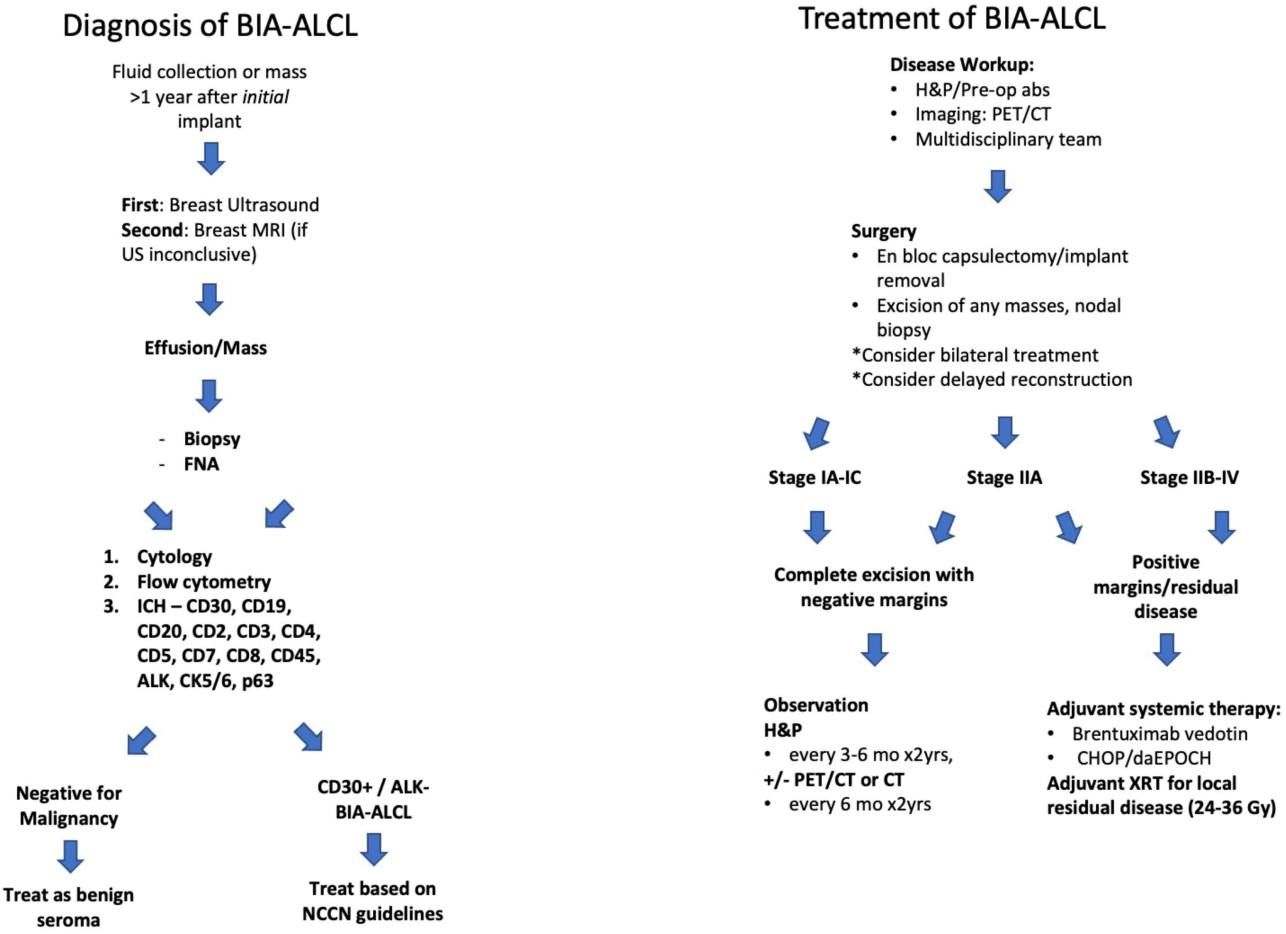
NCCN Guidelines for BIA-ALCL Diagnosis (6).

The NCCN has recently standardized the evaluation of the delayed swollen breast in patients with a history of implants (**Figure 1**) (6). Patients should first be assessed with ultrasound to assess for fluid collection, breast masses, and lymphadenopathy. Complete ultrasound evaluation should include the implant; chest wall; axillary, internal mammary, and supraclavicular lymph nodes; and contralateral breast implant. If ultrasound is equivocal, breast magnetic resonance imaging (MRI) may aid in diagnosis for fluid collections or soft tissue masses. Fine needle aspiration is the standard for sampling periprosthetic fluid collections. Ultrasound guidance is recommended to obtain an appropriate sample and avoid implant injury. Any suspicious masses found during initial imaging should be biopsied and sent for histopathologic analysis. Specimens should be sent for cytology to evaluate cell morphology, immunohistochemistry (IHC) for immune cell markers, and flow cytometry to evaluate cells within the specimen. Cytologic and cell block preparations are utilized to identify neoplastic cells in aspirated effusion fluid.

If an implant-associated malignancy is established, a multidisciplinary team including pathologists, medical and radiation oncologists, surgical oncologists, radiologists, plastic surgeons, and the patient should be leveraged to help stage and treat disease. Pre-operative laboratory studies should include a comprehensive metabolic panel, complete blood count with differential, coagulation studies, and lactate dehydrogenase (6). Hepatitis B testing should be performed if the patient may need chemotherapy. A preoperative positron emission tomography computed tomography (PET/CT) scan is recommended for evaluating associated capsular masses, chest wall involvement, lymph node spread, and organ metastases (8). One must keep in mind that a PET/CT performed for the first three months after surgical intervention is unreliable due to post-operative inflammatory changes within the tissue (6). Treatment modality is determined by the malignancy and extent of disease (**Table 1**).

**Table 1:** Breast implant-associated malignancies – presentation, diagnosis, findings, and treatment.

Malignancy	Worldwide Cases	Presentation	Imaging Diagnostics	Flow Cytometry/ IHC	Pathology	Intraoperative Findings	Treatment	Adjuvant Therapy	Prognosis
BIA-ALCL	1,355 cases	Pain, unilateral swelling	- Diagnostic Ultrasound-Guided FNA - Breast MRI if ultrasound is equivocal - PET/CT after diagnosis is made	CD30 + ALK - CD43 + CD4 + CD3 + CD8 + CD1a - Tdt - Cyclin D1 -	- Pleomorphic anaplastic cells - Large cell nuclei - Prominent nucleoli - Dense chromatin - Hallmark cells	- Intracapsular straw-color, turbid, viscous fluid collection - Mass pebbling of inner surface of capsule	Total capsulectomy, implant removal, extracapsular mass resection	Stage I: No role Stage II-IV: - Adjuvant radiation 24-36 Gy - Adjuvant chemotherapy R-CHOP + brentuximab	2.8% mortality at 1 year 5% mortality at 5 years
BIA-SCC	16 cases	Pain, unilateral swelling, capsular contracture	- Diagnostic Ultrasound-Guided FNA - Breast MRI to evaluate mass - PET/CT after diagnosis is made	CK5 + CK6 + p63 +	- Invasive squamous cell carcinoma or metaplasia - Keratin debris	- Fungated mass - Granulomatous and keratin debris - Tan/yellow capsules - Viscous turbid seroma	Total capsulectomy, implant removal, radical mastectomy	No response to chemotherapy or radiation therapy	43.8% mortality at 6 months
BIA-DLBCL	14 cases	Pain, unilateral swelling, fevers, night sweats	- Diagnostic Ultrasound-Guided FNA - Breast MRI to evaluate mass - PET/CT after diagnosis is made	CD45 + CD20 + CD19 + CD79a + PAX-5 + BCL-6 + EBER + **κ**- or ƛ- light chain restriction	- Giant cell reaction - Pleomorphic lymphoid cells - Atypical nuclei - Heterogenous chromatin pattern	- Tan thickened capsules - Granular, gritty inner lining - Necrotic fibrinoid material	Total capsulectomy, implant removal, extracapsular mass resection	No definitive role for chemotherapy or radiation therapy	No reports of mortality in the literature

## 3 Breast implant associated large cell lymphoma

BIA-ALCL is a CD30-positive T-cell lymphoma that arises around textured breast implants. This disease is distinct from a primary breast lymphoma, which is typically a B-cell lymphoma that arises within the breast parenchyma. The etiology of BIA-ALCL is unknown but likely triggered by chronic inflammation. Implant texturization is indisputably a driver, while host genetic factors, and time likely play a role in tumorigenesis. Bacterial infection and biofilm formation, specifically from *Raltosonia spp*, or perhaps the lipopolysaccharide coat of Gram-negative bacteria was thought to a play a primary role in the pathologic inflammation leading to BIA-ALCL (9). However, more recent research suggests other potential inciting events that may obscure the exact etiologic pathway, including mechanical stress, implant toxins, and surface tribology (10–14). Curiously, a relative attenuation of circulating T-helper cells may occur in the first couple days following placement of a textured, but not smooth breast implant (15). While each of these studies proposes a different “trigger,” chronic inflammation is the common thread, and is the most likely facilitator of malignant transformation to BIA-ALCL (16).

Next generation sequencing performed on patients with BIA-ALCL has often shown activating mutations in the JAK-STAT signaling pathway, most commonly STAT3 and JAK1 (17, 18). BIA-ALCL has also recently been associated with upregulation of hypoxia signaling proteins, specifically carbonic anyhydrase-9 (19). Recently, whole exome sequencing (WES) and whole genome sequencing (WGS) have reported several genomic aberrations associated with BIA-ALCL, but no true driver mutation. These include deletions in chromosome 20q13.13, 20q11.22-q13.2, as well as a critically deleted region on chromosome 11 (11q22.3) corresponding to the ataxia-telangiectasia mutation (ATM) gene (13, 17, 20). Cytokine expression levels also help distinguish BIA-ALCL, which has been characterized by T-helper 2-associated cytokine levels and an IL10 to IL-6 ratio >0.104 (21–23). These findings not only further characterize BIA-ALCL, but open the door for novel treatments and targeted immunotherapies based on expression profile (24, 25).

All verified cases of BIA-ALCL with complete implant history have been exclusively discovered in patients with a history of textured implants, many of which have been recalled, or in some countries banned, due to this association (26–28). Of the 1,130 medical device reports of ALCL, 37 cases were found in patients with smooth implants. However, these patients either previously had textured implants or insufficient implant history (29). By contrast, implant fill (saline or silicone) and reason for implantation (augmentation or reconstruction) has no defined relationship to BIA-ALCL. Therefore, a thorough surgical history including all previous implanted devices should be obtained in all patients presenting with delayed seroma.

**Figure 2:**
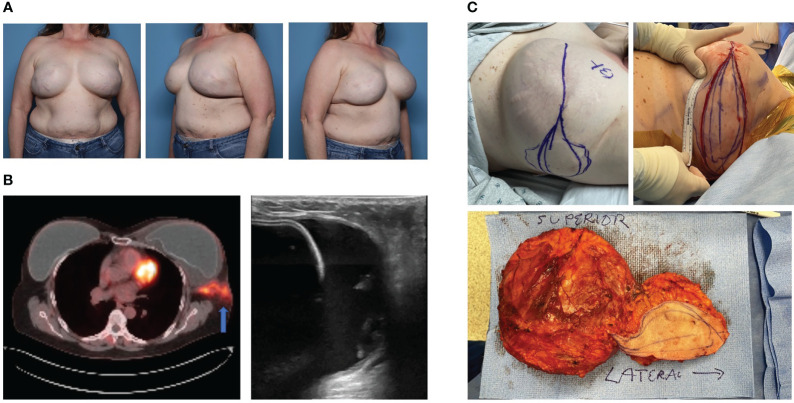
Case of Bilateral Disseminated BIA-ALCL. **(A)** A 52-year-old female with history of left stage 1 breast cancer and right ductal carcinoma *in situ* who underwent neoadjuvant chemotherapy, bilateral mastectomy and implant based breast reconstruction (subpectoral macrotextured breast implants) in 2008. She developed left breast swelling in 2021 and seroma aspirate was consistent with BIA-ALCL. **(B)** PET-CT revealed increased metabolic activity within the left capsule with associated extracapsular involvement into the ipsilateral axilla. Ultrasound revealed intracapsular fluid collection surrounding the implant. **(C)** Bilateral *en bloc* capsulectomies were performed with mass resection extending to the left axilla. Pathology revealed left Stage 4 BIA-ALCL and incidentally found right Stage 1 BIA-ALCL. The patient underwent adjuvant left chest wall radiation and brentuximab vedotin immunotherapy. The patient has had a complete metabolic response to therapy and no evidence of recurrence at 20 months post-operatively.

The incidence of BIA-ALCL in patients with a history of textured implants varies widely, reported as high as 1:355 patients to as low as 1:40,000 patients (26, 30–32). Age of diagnosis is also varied, with an median of 53 years old (ranging 24-90 years old), while time from implantation to diagnosis is consistently prolonged, with an average timeframe of 7-10 years (6, 16, 32, 33). BIA-ALCL manifests as a mass or, more commonly, a rapidly enlarging periprosthetic fluid collection surrounding an implant years after implantation (34). Other local and systemic symptoms reported in patients diagnosed with BIA-ALCL include pain, lymphadenopathy, skin rash, fevers, and capsular contracture (6, 35). In the case of advanced disease, BIA-ALCL typically spreads to the ipsilateral axillary nodes, with supraclavicular, internal mammary, or mediastinal nodal involvement occurring less commonly (36). Intraoperative findings may demonstrate intracapsular periprosthetic fluid collection containing fibrinous material with or without extracapsular spread. Herein, we describe a patient with disseminated BIA-ALCL that presented with a swollen left breast (**Figure 2A**). PET scan reveals T4 lesion with extracapsular disease on the left side (**Figure 2B**), mandating excision of adjacent axillary soft tissues in conjunction with *en bloc* capsulectomy (**Figure 2C**). Importantly, this patient has a prophylactic total capsulectomy on the contralateral right side (ie. entire capsule and breast implant as a single unit, but not adjacent margin of soft tissue) and was noted to have an occult T1 luminal capsular BIA-ALCL. We recommend contralateral prophylactic total capsulectomy in patients undergoing therapeutic *en bloc* capsulectomy because of a 1-3% risk of bilateral disease (6, 37–39).

The diagnosis of BIA-ALCL is made through cytologic and immunohistochemical analysis of periprosthetic fluid. The neoplastic cells of BIA-ALCL are large, pleomorphic cells with anaplastic morphology. The cell nuclei are large, oval or multilobulated, with dense chromatin, and have prominent nucleoli and frequent mitoses (40). Commonly described in association with ALCL are the “hallmark” cells, which have horseshoe- or kidney-shaped nuclei and are found in a majority of cases (41). BIA-ALCL is universally CD30 positive and ALK negative. Expression of CD30 is a fundamental finding in BIA-ALCL; however, it is important to note CD30 expression alone is not diagnostic for BIA-ALCL, as it is non-specifically expressed by benign inflammatory cells (6, 42–44). Likewise, because other forms of ALCL also lack ALK expression, ALK negative IHC alone does not establish a diagnosis of BIA-ALCL. Additional biomarkers may be required to establish the diagnosis and exclude other malignancies, including CD2, CD3, CD4, CD5, CD7, CD8, and CD45 expression.

The Lugano modification of the Ann Arbor staging system is traditionally used to stage all forms of Non-Hodgkin lymphoma (45). However, nearly all BIA-ALCL cases are staged as Stage IE – lymphoma with single extranodal involvement, or Stage IIE – extranodal disease with local nodal involvement. Further, this staging system does not account for capsular involvement (46). Thus, MD Anderson Cancer Center developed a tumor, lymph node, metastasis (TNM) solid tumor staging system that was adapted by the NCCN to further characterize BIA-ALCL (**Table 2**). While many reported cases are categorized as stage I, over 25% of BIA-ALCL cases have extracapsular involvement at the time of diagnosis (6, 33, 46). This staging system has demonstrated improved efficacy in predicting survival and recurrence compared to the Ann Arbor staging system (46).

**Table 2:** – BIA-ALCL Tumor, Lymph Node, Metastasis (TNM) Classification and Staging (6) TNM Staging.

			TNM Staging
T: Tumor Extent		IA	T1 N0 M0
T1	Confined to effusion or inner capsule layer	IB	T2 N0 M0
T2	Early capsular infiltration	IC	T3 N0 M0
T3	Cell aggregates or sheets invading capsule	IIA	T4 N0 M0
T4	Lymphoma infiltrates beyond capsule	IIB	T1-3 N1 M0
N: Lymph Nodes		III	T4 N1-2 M0
N0	No nodal involvement	IV	Tx Nx M1
N1	Disease in regional lymph node		
N2	Disease in multiple regional lymph nodes		
M: Metastasis			
M0	No distant spread		
M1	Spread to other organs/distant lymph tissue		

Following diagnosis and pre-operative imaging, the mainstay of treatment for BIA-ALCL is total capsulectomy (**Figure 2C**) (6, 46). There is no proven role for mastectomy, sentinel lymph node biopsy, or axillary node dissection (6). Surgery should always be conducted with an *en bloc* capsulectomy to remove the implant in continuity with the capsule, any associated extracapsular masses, and a margin of contiguous healthy tissue. Complete surgical excision with negative margins is associated with long-term, disease free survival, and may be adequate for disease localized to the capsule (Stage IA-IIA) (46, 47). However, disease recurrence is nearly 3-fold higher in Stage II and Stage III disease, and is more likely with incomplete resection, partial capsulectomies, or positive margins.

The use of adjuvant therapy for BIA-ALCL is limited to patients with residual or disseminated disease. The NCCN recommends radiation therapy of 24 to 36 Gray (Gy) for any local residual disease or unresectable masses due to chest wall involvement (6). In patients with disseminated disease, first-line systemic therapy should include the combination regimen CHOP (Cyclophosphamide, Adriamycin, Vincristine, and Prednisone) and brentuximab vedotin, a CD30 monoclonal antibody that has recognized survivability benefits when treating peripheral T-cell lymphomas (48–52).

Worldwide, there have been a total of 59 reported deaths related to BIA-ALCL up until April 1, 2022, with more under review (33). Overall survivability of early stage BIA-ALCL is reported as 94% at 3 years and 91% at 5 years, respectively (46). Later stage of presentation is associated with decreased survivability and higher risk of recurrence (46). Patients who reach remission should be monitored for recurrence every 3 to 6 months for 2 years, and radiologic imaging with CT or PET scan should be considered every 6 months for 2 years due to the high 3-year recurrence risk at all stages (6).

## 4 Breast implant associated squamous cell carcinoma

Breast implant associated squamous cell carcinoma (BIA-SCC) is a rare but aggressive malignancy that originates from the breast implant capsule. This entity was first proposed by Paletta and colleagues in 1992, who reported a case of BIA-SCC in a patient who underwent breast augmentation with silicone implants 16 years prior (53). Since this initial case report nearly 30 years ago, there have been 16 verified cases of BIA-SCC according to the American Society for Plastic Surgeons (ASPS), with more cases under review including one we present herein (34, 54–59). Though rare, this malignancy has garnered attention recently due to its aggressive nature and high mortality.

The origin of the squamous cell epithelium in this malignancy is unclear. Similar carcinogenic processes have been described with foreign bodies in other tissues, including bullet wounds and dental or orthopedic implants (60–63). It is proposed that ductal epithelium can be displaced at the time of pocket implantation, resulting in squamous epithelialization of the breast implant capsule (54, 55, 59). Additionally, macrophages and lymphocytes infiltrate the breast pocket and release cytokines to wall-off the foreign body. This may become exaggerated over a protracted course, such as in the case of a permanent breast implant. It is well-known that chronic inflammation can lead to an imbalance in inflammatory and apoptotic cell signaling pathways, resulting in tissue metaplasia (64). Such is the case for intestinal metaplasia of the esophagus and stomach in response to chronic acid exposure. Similar concepts may be applied to BIA-SCC: chronic inflammation and fibrosis surrounding the breast implant can inadvertently stimulate metaplastic squamous epithelium production within the capsule, a precursor to squamous cell carcinoma.

Age of diagnosis is highly variable, ranging from 40-81 years old (34, 54–59). The time interval from initial breast implant surgery to BIA-SCC diagnosis ranges from 11-41 years, and while consistently protracted, does overlap to an extent with the timeframe during which BIA-ALCL may develop. This dysplastic process, which may be indolent in nature, should therefore remain on the differential diagnosis along with BIA-ALCL when assessing patients who present with new breast swelling in the context of a breast implant multiple years after implantation.

Presentation of BIA-SCC includes unilateral breast pain, erythema, and fluid collection. Patients may also present with some degree of capsular contracture and implant malposition, though this is not a uniform finding at time of diagnosis. Of the 16 reported cases, eleven occurred following breast augmentation and five occurred following breast reconstruction (34, 54–59, 65). Cases have been reported in all implant types, including saline and silicone implants with either textured or smooth surfaces (54–59, 65, 66). However, in a majority of these reports, implant surface and device history are not reported and so there is insufficient information to determine whether it has etiologic relevance. Future cases including completely documented device history will help determine if an association with implant texturing exists.

**Figure 3:**
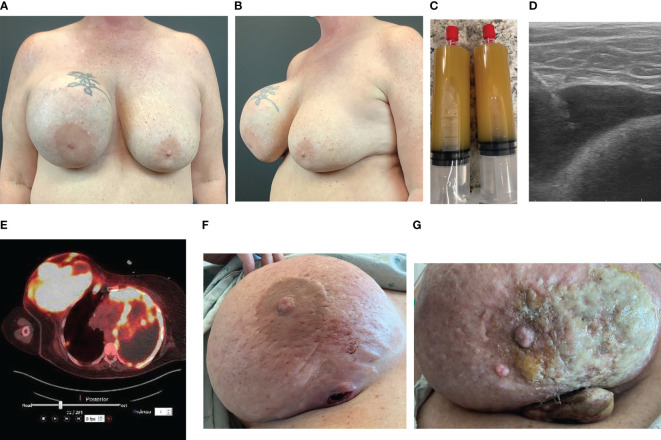
Case of BIA-SCC. **(A)** Anterior view of a 56-year-old female with history of macrotextured breast implant in 2006 presented with unilateral right breast swelling, pain, and capsular contracture 16 years after implantation. **(B)** Oblique view at presentation. **(C)** In-office seroma aspirate in May 2022 revealed copious amount of yellow, turbid fluid. Cytology and IHC analysis demonstrated acute inflammation and abundant squamous cells, but no malignant cells. BIA-ALCL workup negative. **(D)** Ultrasound shows fluid collection between implant and capsule. This patient then underwent bilateral implant removal with total capsulectomy. Intraoperative findings revealed thickened capsule with an associated tan-pink, indurated, nodular mass. Pathology revealed poorly differentiated SCC. The patient was placed on adjuvant pembrolizumab, abraxane, and carboplatin chemotherapy. **(E)** PET/CT 3 months post-implant removal revealed increased metabolic uptake throughout the right breast and axilla, and left chest wall and pleura, consistent with metastatic SCC. **(F)** By September 2022, the primary malignancy began to erode through the patient’s breast. **(G)** One month later the primary malignancy progressed despite chemotherapy. She expired weeks later from complications related to pleural metastases.

The ASPS recommends FNA and cytology of any delayed seroma prior to surgical intervention (67). Most cases reported in the literature have not involved FNA of seroma fluid prior to operative intervention, as many of these cases were reported prior to new diagnostic recommendations (54–59, 65, 66). Seroma aspirates confirming BIA-SCC express epithelial carcinoma marker CK 5/6 and squamous cell transcription factor p63, and contain squamous cells and keratin. We report a case of a patient presenting with unilateral breast swelling 16 years following cosmetic breast augmentation with a macrotextured implant (**Figures 3A, B**). To properly evaluate for BIA-SCC, seroma fluid aspirate (**Figure 3C**) should be sent for IHC looking for CD30 and ALK to evaluate for BIA-ALCL, along with CK 5/6 and p63. Flow cytometry should be employed to look for T-cells, squamous cells, and keratin. One should note that seroma aspirate is not completely comprehensive for detecting malignancy, and tissue biopsy is commonly required to achieve diagnosis. For the case we present herein, initial seroma aspirate failed to reveal malignancy; squamous cell carcinoma was only found on pathology after complete capsulectomy was performed.

Pre-operative imaging facilitates surgical planning. Ultrasound is commonly used to guide aspiration of seroma fluid for analysis (**Figure 3D**). Breast MRI with and without contrast can be employed to identify any masses. Findings consistent with BIA-SCC will demonstrate an ill-defined mass arising from the breast capsule, with possible extent into the chest wall. PET-CT should be employed prior to intervention to appropriately determine extent of disease (**Figure 3E**).

Intraoperative findings of BIA-SCC include fungating breast capsule masses with granulomatous and keratinized debris contained within a viscous, turbid seroma fluid. In a majority of reported cases, this malignancy arises from the posterior aspect of the implant capsule, with spread of keratinaceous material into the pectoralis muscle and axillary tissue (**Figures 3E–G**) (53–57, 59). Capsules are commonly found intact with a thickened appearance and yellow hue. Histology from these capsules and associated granulomatous material demonstrates invasive keratinized squamous cell carcinoma and metaplasia with evidence of acute on chronic inflammation. These findings further support the theory that chronic inflammation stimulates malignant transformation.

The overall prognosis for BIA-SCC is grim, with a 6-month mortality rate of 43.8% (67). Early diagnosis and treatment can have life-lengthening benefits. Current treatment recommendations are likely to evolve as more diagnoses are described. The current treatment recommendation is surgical with *en bloc* capsulectomy and radical mastectomy (67). Surgeons should be aggressive, and should not hesitate to resect chest wall or axillary contents if there is suspicion for invasive malignancy. This is of utmost importance when treating BIA-SCC, as incomplete resection is associated with aggressive recurrence and increased mortality. There does not appear to be a role for adjuvant chemotherapy or radiotherapy, as the malignancy has thus far demonstrated little to no therapeutic response to either treatment modality.

## 5 Breast implant associated diffuse large B-cell lymphoma

**Figure 4:**
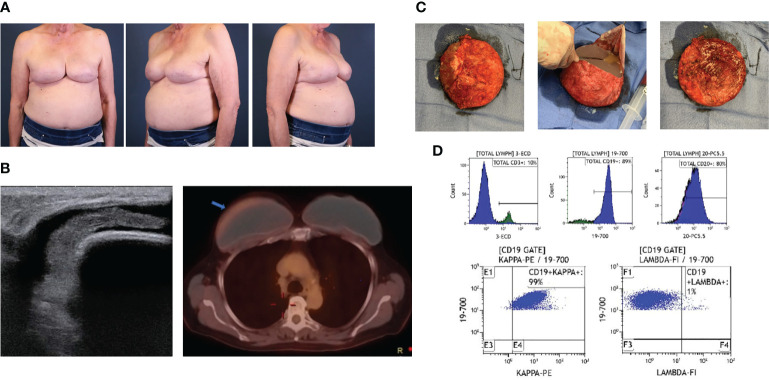
Case of BIA-DLBCL. **(A)** A 71-year-old female with history of left ductal carcinoma *in situ* status post bilateral mastectomies and immediate microtextured breast implant reconstruction in 2015 presented with unilateral right breast swelling three years after implantation with seroma aspirate consistent with BIA-DLBCL. **(B)** Ultrasound demonstrated peri-implant fluid collection and PET/CT revealed minimally increased metabolic uptake around the right breast capsule. **(C)** Bilateral *en bloc* capsulectomies were performed, demonstrating thick implant capsules and yellow-tinged intracapsular fluid collection. **(D)** Flow cytometry from the right breast capsule revealed a majority of cells expressing CD19 and CD20 with kappa light-chain restriction. No additional adjunct therapies were required. She has had a complete response and has no evidence of recurrence at 3 years post-operatively.

Lymphomas associated with breast implants are rare and commonly have a T-cell origin. In a small minority of cases, though, delayed unilateral breast swelling years after breast implant placement (**Figure 4A**) have been attributed to other lymphomas. Breast implant associated-B-cell lymphomas are characterized by a more heterogenous cellular origin that includes diffuse large B-cell lymphoma, follicular lymphoma, primary cutaneous lymphoma, intravascular large-cell lymphoma, splenic marginal zone lymphoma, and plasmablastic lymphoma (68–82). Of these diagnoses, breast implant associated diffuse large B-cell lymphoma (BIA-DLBCL) has been most commonly described in the literature, is associated with a delayed seroma (**Figure 4B**), and has been associated, in several instances, with Epstein Barr Virus (EBV).

**Figure 5:**
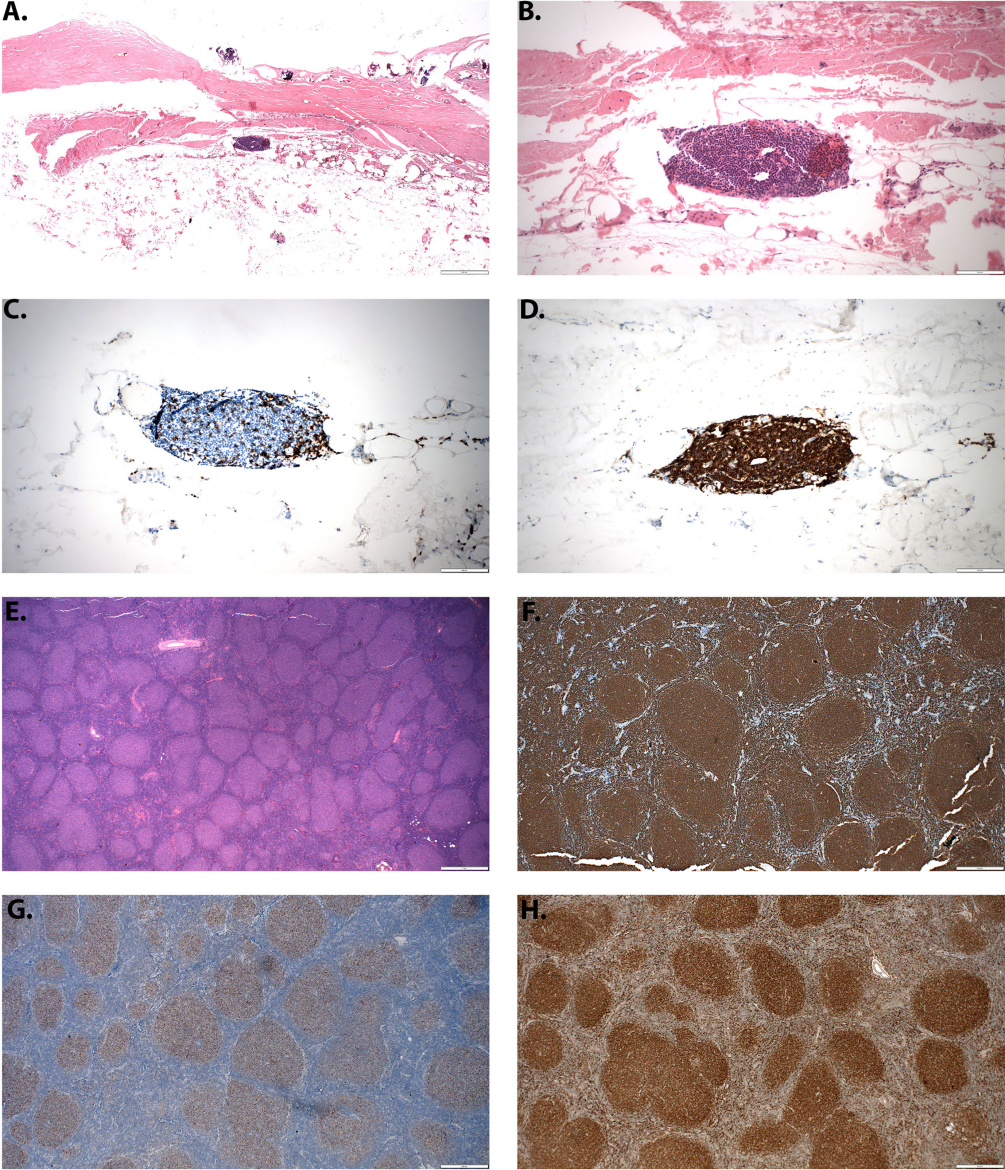
Follicular cell lymphoma associated with breast implant capsule. **(A)** Lymphoid aggerate seen in breast capsule (2x magnification, H&E stain). **(B)** High power view of the aggerate shows atypical lymphocytes with irregular contours (20x magnification, H&E stain). **(C)** A CD3 stain shows few T-cells (20x magnification). **(D)** The atypical lymphocytes are CD20+ B-cells and comprise nearly all of the lymphocytes (20x magnification). **(E)** An adjacent lymph node shows closely packed and expanded follicles without mantle zone consistent with grade 1-2 follicular lymphoma (4x magnification). **(F)** A CD20 stain shows that follicles are composed of B-cells (4x magnification). **(G)** The neoplastic follicles are BCL2 positive (4x magnification). **(H)** BCL6 is positive in follicles (4x magnification).

Due to the broad array of reported B-cell lymphomas, patient presentation is wide-ranging. Of the 28 reported cases, a majority of patients reported breast pain and swelling or palpable mass, while fewer presented with capsular contracture, B symptoms, hepatosplenomegaly, and lymphadenopathy (68–83). Herein, we present two cases, one of BIA-DLBCL (**Figure 4**) and one of breast implant associated follicular lymphoma (**Figure 5**). Age and time from initial implantation to diagnosis ranges from 34 – 83 years old and 6 – 44 years, respectively. Despite these differences, there are similarities, most notably the association with textured, silicone breast implants (70, 81–83).

There have been fourteen reported patients with diffuse B-cell lymphoma, twelve of which were found to be positive for Epstein-Barr Virus. EBV-positive DLBCL has been implicated in states of immunosuppression and chronic inflammation, categorized by the WHO as diffuse large B-cell lymphoma associated with chronic inflammation (DLBCL-CI), of which pyothorax-associated lymphoma is the prototype (84). The presence of longstanding chronic inflammation associated with an indwelling implant may result in proliferation of EBV-transformed B-cells, such as in the case of DLBCL-CI (85). However, the fibrinous material associated with BIA-DLBCL lends itself to a diagnosis similar to fibrin-associated DLBCL (FA-DLBCL), an indolent form of EBV-positive large B-cell lymphoma that has been categorized by the WHO as a clinically distinct subtype of DLBCL-CI (86). This form of lymphoma has been reported in association with pathologic debris surrounding atrial myxomas, endovascular graft thrombi, metallic prosthesis, and pseudocysts. These cases have a much more favorable prognosis in comparison to DLBCL-CI, and may even represent a form of EBV-positive lymphoproliferative disease rather than a lymphoma (87). However, not all cases of implant-associated DLBCL are EBV-positive, including the case we present in **Figure 4**. Further case collection and pathologic investigation is required to characterize this novel lymphoma.

Complete physical evaluation and diagnostic workup for the delayed swollen breast should be obtained to characterize this malignancy. Most cases are localized to the implant capsule, though few have been found to be invasive, mass-forming lymphomas. Gross pathologic findings of DLBLCL exhibit tan, thickened implant capsules with granular, gritty inner lining following *en bloc* capsulectomy (**Figure 4C**). Reported microscopic examination demonstrates focal foreign body giant cell reactions and lymphoplasmacytic aggregates of pleomorphic lymphoid cells, which may have atypical nuclei with numerous mitotic figures, heterogeneous chromatin pattern, and/or prominent nuceloli (21, 70). Immunohistochemical analysis exhibits a wide array of B-cell expression profiles, with inconsistent staining for B-cell markers CD20, CD19, CD79a, PAX-5, and BCL-6 (**Figure 4D**) (70, 72, 83).

*En-bloc* capsulectomy (**Figure 4C**) and implant removal has proven to be adequate in treating localized breast implant-associated B-cell lymphomas (81, 82). We have also used this approach to manage a case of follicular cell lymphoma associated with a breast implant capsule. Distinguishing this case as a follicular cell lymphoma were the findings of lymphoid aggregates seen in the breast implant capsule (**Figure 5A**), atypia with irregular contours (**Figure 5B**), few T-cells (**Figure 5C**), predominance of CD20+ B-cells (**Figure 5D**), closely packed and expanded follicles without mantle zones (**Figure 5E**), that were comprised of B-cells (**Figure 5F**). Follicles were BCL2 (**Figure 5G**) and BCL6 (**Figure 5H**) positive. Given the few case reports and overall indolent nature of the disease, a consensus on the need for adjuvant chemoradiation or radiologic disease monitoring has not been reached. Regular breast imaging has been advocated to evaluate disease recurrence. Long-term follow up data is needed to determine disease prognosis and survivability benefit for each of these treatment modalities.

## 6 Benign delayed seroma

Benign delayed seromas are usually defined as serous fluid collections that develop around an implant more than one year after implantation. In accordance with the NCCN guidelines (6), these are diagnosed primarily by ultrasound, but breast MRI may be required in equivocal cases or when a greater level of sensitivity and specificity are warranted. It has been theorized that benign delayed seromas are the result of trauma, hematoma, subclinical infection, or implant rupture, though they can also occur without an identified precipitating cause. It has been theorized that benign delayed seromas are the result of trauma, hematoma, subclinical infection, or implant rupture, though they can also occur without an identified precipitating cause. Benign delayed seromas are rare events, occurring in less than 1% of subjects in large multicenter trials (88–90).

Prior to the discovery of BIA-ALCL, a majority of early case reports describing delayed seromas occurred in patients who previously had macrotextured breast implants (12, 89, 91, 92). It is conceivable that early case reports of delayed seromas were undiagnosed BIA-ALCL, as all diagnosed patients had a history of textured implants. It is important to note that up to 10% of delayed fluid collections associated with breast implants are malignant on further diagnostic evaluation (42, 88, 89). Though similar in physical presentation, the pathogenesis of benign delayed seromas and BIA-ALCL are distinct. BIA-ALCL develops as a malignant effusion due to increased vascular permeability resulting from cellular production of interleukins and elevated oncotic pressure caused by the high cellularity and protein content of the fluid. Benign delayed seromas have a wide variety of purported etiologies, including an idiopathic one. Though associated with trauma and subclinical infection, benign delayed seromas typically lack a microbiologic or cytologic biomarker (92). The detection of CD30 positive cells on IHC aids the diagnosis of BIA-ALCL and helps differentiate a benign delayed seroma from a malignant one, drastically changing overall management (43).

Treatment of benign delayed seroma varies based on surgeon and patient preferences, ranging from serial aspirations to complete capsulectomy. In their multicenter retrospective review of delayed seromas, Spear et al. expounded upon a graduated approach to treating delayed seromas that included antibiotics, serial aspirations, drain placement, and surgical resection (90). A majority of patients required surgical intervention to reach full resolution, while 28.5% of patients were able to be successfully managed with aspiration or antibiotics alone. In their series, all aspirate cultures were negative for planktonic bacteria identifiable with standard culture techniques. They did not perform advanced biofilm detection techniques such as 16S rRNA sequencing, immunohistochemistry for bacteria-specific probes, or scanning electron microscopy. Likewise, routine CD30 immunohistochemistry testing was not performed as BIA-ALCL was not a well-known diagnosis at this time. We believe a similar algorithm can be used to treat delayed seromas once they are determined to be benign and non-infectious.

## 7 Double capsule

**Figure 6:**
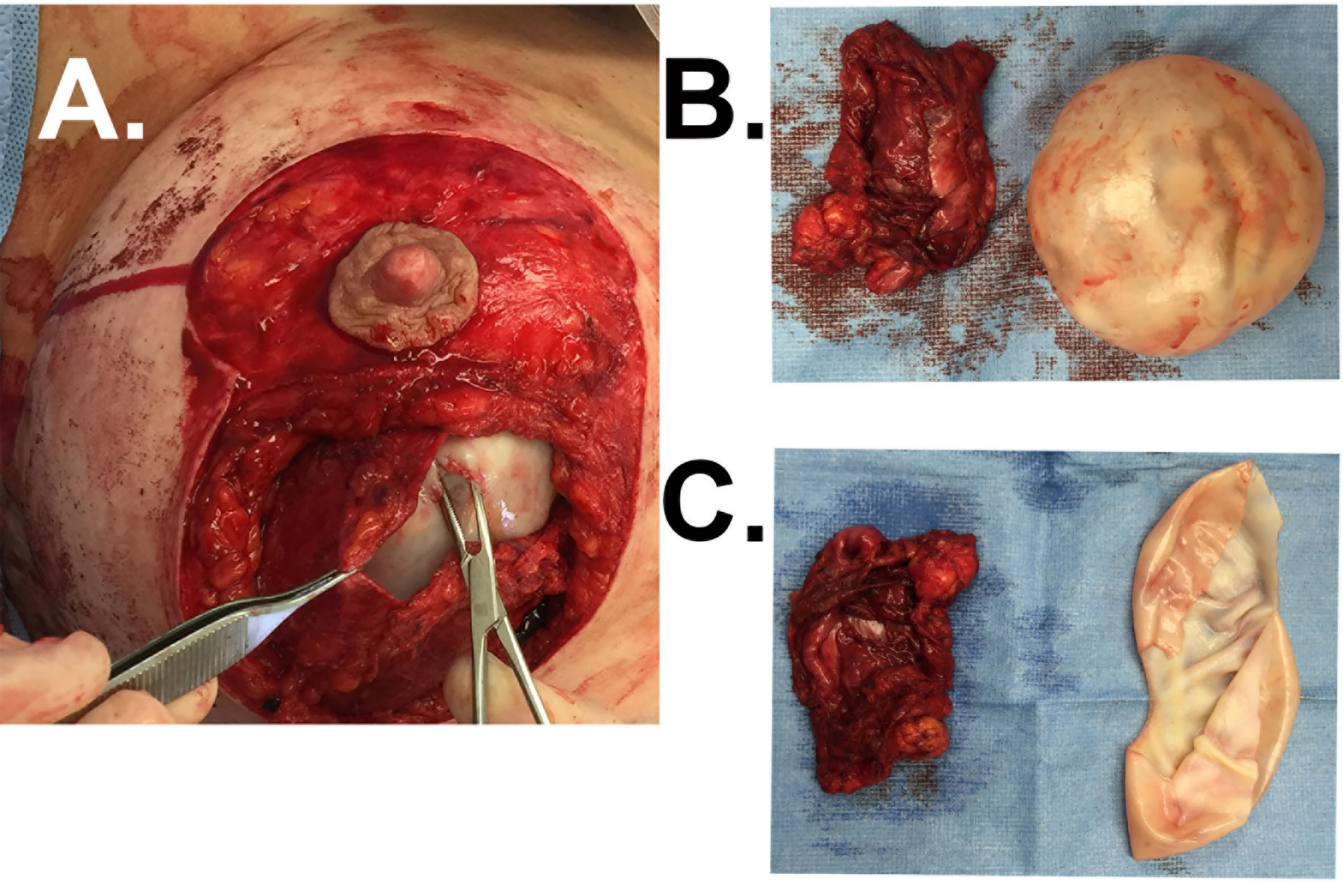
Case of double capsule. **(A)** Patient with a macrotextured saline breast implant placed for cosmetic reasons 7 years prior presents with a rapidly developing seroma of the right breast. The forcep reflects the outer capsule and the hemostat penetrates the inner capsule that is intimately associated with the implant. Clear fluid was identified between these two layers *in situ*. **(B)** The outer capsule is free from the inner capsule that is *in continuity* with the textured implant shown here. **(C)** The inner capsule now dissected free from the implant surface.

Another rare but benign etiology of delayed breast swelling is the double capsule. This occurs when the inner capsule envelope adheres to the implant surface while a distinct outer capsule adheres to surrounding tissues, divided by an intercapsular space that may contain a seroma (**Figure 6A**) (11, 12). Typically, the outer capsule can be dissected from the surrounding soft tissues while the inner capsule remains intimately associated to the textured device (**Figure 6B**) unless it is deliberately peeled off the implant (**Figure 6C**). Hall-Findlay was the first to report on her experience with double capsule formation (12). Initial patient presentation is highly variable, including persistent seroma, capsular contracture, bottoming out, and asymmetry (12, 93, 94). Similar to BIA-ALCL, this phenomenon primarily develops in patients with textured implants. It is theorized that macrotextured surfaces induce some adherence of the capsule to the implant, which can result in mechanical shearing of the implant capsule from the implant surface and seroma formation (93, 95). Bacterial biofilms may contribute to double capsule formation by weakening capsule strength and facilitating extracellular matrix delamination and double-capsule formation (96). Diagnostic evaluation should follow the NCCN guidelines. The only surgical modality efficacious in treating double capsule includes inner capsulectomy with or without outer capsulectomy and smooth implant exchange (12, 94).

## 8 Infection

When examining the delayed swollen breast, implant infection must be taken into consideration. Reported incidence of implant infection ranges from 0-2.5% following breast augmentation, and up to 35% following breast reconstruction after mastectomy (97–100). Acute infections typically occur within weeks to months following implant placement. Patients commonly present with breast pain, drainage, and erythema, and systemic symptoms including fevers, nausea, and vomiting. Possible sources of infection include the patient’s skin or breast microbiota, contaminated implant or irrigation fluid, surgical manipulation, and hematogenous spread.

In addition to causing acute infections, many of these bacteria have evolved to adhere to implant surfaces, forming assemblages of surface-adherent bacteria encapsulated in extracellular polymers known as biofilms. The formation of these biofilms around an implant are implicated in subclinical infections, capsular contracture, and other systemic symptoms. Subacute infections, which can occur months to years after surgery, have a more indolent course, making them more difficult to distinguish from other diagnoses. Patients may present with chronic pain, persistent swelling and drainage, wound healing problems, or implant migration. Hematogenous spread of bacteria from distant sites play a crucial role for developing late onset breast implant infections.

Initial evaluation for breast implant infection should rely heavily on the patient history and physical exam findings. Full history of recent illnesses or infections and surgical interventions should be reviewed. Providers should look for subtle signs of infection including fevers, nausea and vomiting, and new breast pain, erythema, or drainage. Laboratory tests should include a comprehensive metabolic panel and complete blood count. Diagnostic evaluation for subacute infections should include complete breast ultrasound to evaluate for drainable fluid collections, cultures, and bacterioscopic smear test may be considered to confirm and characterize infection. Malignancy should also be ruled out with imaging should new breast masses or lymphadenopathy be identified, and FNA performed of seroma fluid with histologic examination. While some investigators report successfully salvaging periprosthetic implant infections using negative pressure therapy with or without irrigation (101), treatment traditionally warrants surgical washout and implant explantation.

## 9 Traumatic hematoma

Early hematoma directly following breast implant placement, whether it be for reconstructive or aesthetic purposes, is a well-documented post-operative complication occurring in 0.6-10.3% of all cases (102–104). Peri-prosthetic late hematomas that occur more than 6 months after surgery are considered a rare complication, many of which have unknown causes. Chest trauma is an acute inciting factor that can result in spontaneous capsular sheering and hematoma formation. Likewise, chronic inflammation or systemic therapies, such as corticosteroids, chemotherapy, or systemic anticoagulation, can damage peri-capsular arteries and lead to late capsular hematoma (105–107). In the absence of a clear inciting event, it is thought that mechanical friction between the prosthesis and the highly vascular capsule, with a consequent capsule microfractures, may play a role in delayed hematoma (104). In evaluating patients for delayed hematoma, MRI and ultrasonography may be performed, but are not helpful in distinguishing hematoma from implant rupture, and may lead to false positives. Treatment includes hematoma evacuation and implant exchange with or without capsulectomy in patients who want to maintain their breast size. Hematoma is also a risk factor for the development of capsular contracture and should be adequately addressed to avoid this latent complication.

## 10 Breast cancer

With nearly one in eight women diagnosed with breast cancer within their lifetime, the possibility that a new breast mass or fluid collection in a patient with breast implants is related to primary or recurrent breast cancer can occur (108). Thus, physicians must have a high index of suspicion for breast cancer when evaluating the delayed swollen breast. In particular, invasion of dermal lymphatics in inflammatory breast cancer can lead to rapid swelling, erythema, and pitting edema thus presenting as delayed swelling of the breast in a women with previous implant-based breast augmentation (108). Therefore, all patients who present with delayed breast swelling should undergo diagnostic mammography, breast MRI, and/or complete breast ultrasound to evaluate for cancerous lesions (109–111). Breast MRI is an important imaging modality that can be employed in younger patients with highly dense breast tissue. Any suspicious masses or should be evaluated by a radiologist and surgical oncologist to determine need for further evaluation and treatment.

## 11 Other considerations

In addition to complications associated with breast implants, patient medical comorbidities must be taken into consideration when evaluating the swollen breast. Previous case reports have demonstrated that unilateral breast edema may be a manifestation of congestive heart failure, specifically in elderly patients (112–114). Patients will present with signs and symptoms of heart failure on physical exam, including jugular venous distension, pretibital pitting edema, and pulmonary congestion. Chest radiographs will demonstrate cardiomegaly and pulmonary edema, and diagnosis will be made by decreased ejection fraction on electrocardiography.

## 12 Conclusion

The delayed presentation of a swollen breast in patients with a history of breast implants is a diagnostic challenge to all physicians. Though many cases are benign, one must carefully follow the NCCN guidelines to properly evaluate for the malignancy, including BIA-ALCL, BIA-SCC, and BIA-DLBCL, and recurrent or new primary breast cancer. All cases of malignancies associated with breast implants should be reported to the FDA’s Manufacturer and User Facility Device Experience (MAUDE) database and the device manufacturer. To improve our understanding of these rare cancers, cases of breast implant malignancy from the United States should be reported to the PROFILE registry (https://plasticsurgery.formstack.com/forms/profile_case_submission) and equivalent registries in other countries. Moreover, genomics continue to play a critical role in the diagnosis and identification of targeted therapies to more effectively manage both breast cancers and breast-implant associated malignancies (24, 25). Further, though these malignancy are rare, all patients receiving breast implants should be counseled pre-operatively on the risk of each of these cancers, and particularly BIA-SCC due to its severity and mortality (67).

## Author contributions

TM takes full responsibility for the integrity and accuracy of this review articles. All authors approve the final article and agree to be accountable for all aspects of the work. Concept and design: GK, AK, TM. Drafting of the manuscript: GK, AK, TM. Critical revisions of the manuscript for important intellectual content: all authors. Supervision: TM.
